# Integrated self-regulating resistive heating for isothermal nucleic acid amplification tests (NAAT) in Lab-on-a-Chip (LoC) devices

**DOI:** 10.1371/journal.pone.0189968

**Published:** 2017-12-21

**Authors:** Tamas Pardy, Indrek Tulp, Clemens Kremer, Toomas Rang, Ray Stewart

**Affiliations:** 1 Selfdiagnostics Deutschland GmbH, Leipzig, Saxony, Germany; 2 Thomas Johann Seebeck Institute of Electronics, Tallinn University of Technology, Tallinn, Estonia; 3 Bay Materials LLC (in commercial partnership with Heatron LLC), Fremont, California, United States of America; Imperial College London, UNITED KINGDOM

## Abstract

Isothermal nucleic acid amplification tests (NAAT) in a Lab-on-a-Chip (LoC) format promise to bring high-accuracy, non-instrumented rapid tests to the point of care. Reliable rapid tests for infectious diseases allow for early diagnosis and treatment, which in turn enables better containment of potential outbreaks and fewer complications. A critical component to LoC NAATs is the heating element, as all NAAT protocols require incubation at elevated temperatures. We propose a cheap, integrated, self-regulating resistive heating solution that uses 2xAAA alkaline batteries as the power source, can maintain temperatures in the 60–63°C range for at least 25 minutes, and reaches the target range from room temperature in 5 minutes. 4 heating element samples with different electrical characteristics were evaluated in a thermal mock-up for a LoC NAAT device. An optimal heating element candidate was chosen based on temperature profiling. The optimal candidate was further evaluated by thermal modelling via finite element analysis of heat transfer and demonstrated suitable for isothermal nucleic acid amplification.

## 1 Introduction

Novel developments in Point-of-Care and Lab-on-a-Chip devices are targeted at applications that require temperature control, especially nucleic acid amplification tests (NAAT). NAATs are the current gold standard in pathogen detection with PCR (polymerase chain reaction) as the most well-known and widely practiced example. Isothermal NAATs require a single temperature range to be maintained in the reaction volume for the specified amplification time [1–4], making them more suitable for Lab-on-a-Chip applications than PCR, which requires thermal cycling. In the ideal case, a LoC NAAT device would be non-instrumented, self-contained and preferably disposable. However, non-instrumented LoC NAAT devices pose unique technical challenges resulting from space, power and cost constraints characteristic of the platform [5]. These constraints have made commercialization difficult in the past, thus limiting availability to the general public.

Most reported Lab-on-a-Chip devices are instrumented and rely on external means for temperature control, such as Peltier cells [6–8] or pre-heated liquids [9] introduced via channels surrounding the reaction volume. Electrical heating is the primary candidate for integrated temperature control in Lab-on-a-Chip systems, however, space and power constraints pose unique technical challenges to overcome. Most works published to date on integrated heating options have been resistive thin film heating elements [10–14], or integrated micro-Peltier cells, typically controlled externally, resulting in a minimally-instrumented device rather than a self-contained one. Integrated self-regulating heating would be an ideal candidate for non-instrumented NAATs [15–19], but has not become widespread yet due to the aforementioned technical limitations of space and power. Furthermore, per-device cost is a factor significantly limiting the development of disposable rapid tests that rely on electrical temperature control. Another option is offered by chemical heating [20–22], which would be advantageous for a non-instrumented setup due to its low cost, but typically has a larger footprint than electrical heaters and is challenging to regulate. Exothermic chemical reactions have a highly non-linear heat release profile in time, which is compensated by using phase-change materials (PCM) that carry away the excess heat. The added design complexity results in a longer development time and higher risk, making chemical heaters unfavorable for commercial LoC devices. Therefore, cheap, self-regulating resistive heating elements would be an ideal candidate for commercial LoC NAATs, including single-use, disposable devices.

We propose a temperature control solution based on self-regulating resistive heating that has a temperature control precision sufficient for loop-mediated isothermal nucleic acid amplification (LAMP), is cheap enough to be disposable and power-efficient enough to run from 2xAAA alkaline batteries. Furthermore, it can reach target temperature in 5 minutes and hold for 25 minutes. We demonstrate a heating element candidate chosen from 4 commercial prototype samples subjected to electrical and thermal evaluations. The optimal candidate is then analyzed in a thermal mock-up of a LoC NAAT system, as well as thermally modelled via finite element modelling to demonstrate its functionality.

## 2 Materials and methods

### 2.1 Heating element

Self-regulating resistive heating elements are electrical heaters that are capable of maintaining their set temperatures without any external regulating electronics, regardless of ambient temperatures [15]. They are composed of materials that have a positive temperature coefficient of resistance (PTCR), which means the heating element will exhibit a rapid rise in resistance at elevated temperatures. This limits the input current passing through the element. The design parameters of these heating elements must be precisely set to match the desired set temperature. Self-regulating heaters are inherently advantageous for Lab-on-a-Chip and Point-of-Care applications that require temperature control, as they can be designed to have a small footprint, can be made power-efficient, and are typically cheap enough to be suitable for single-use, disposable rapid tests.

For the proposed heating element in this paper, the target temperature was defined as 61.5 ± 1.5°C, which fell into the target range (60–65°C [23]) required by the loop-mediated isothermal amplification (LAMP) assay. The heater was expected to reach target under 5 minutes and hold it for over 30 minutes. Furthermore, driving voltage was specified as 3V_DC_, with 2xAAA alkaline batteries as the power supply in mind. AAA alkaline batteries were chosen for their low price, relatively small size and comparably good energy density. In addition, they do not generate hazardous waste and recycling options are also available for them.

**Fig 1:**
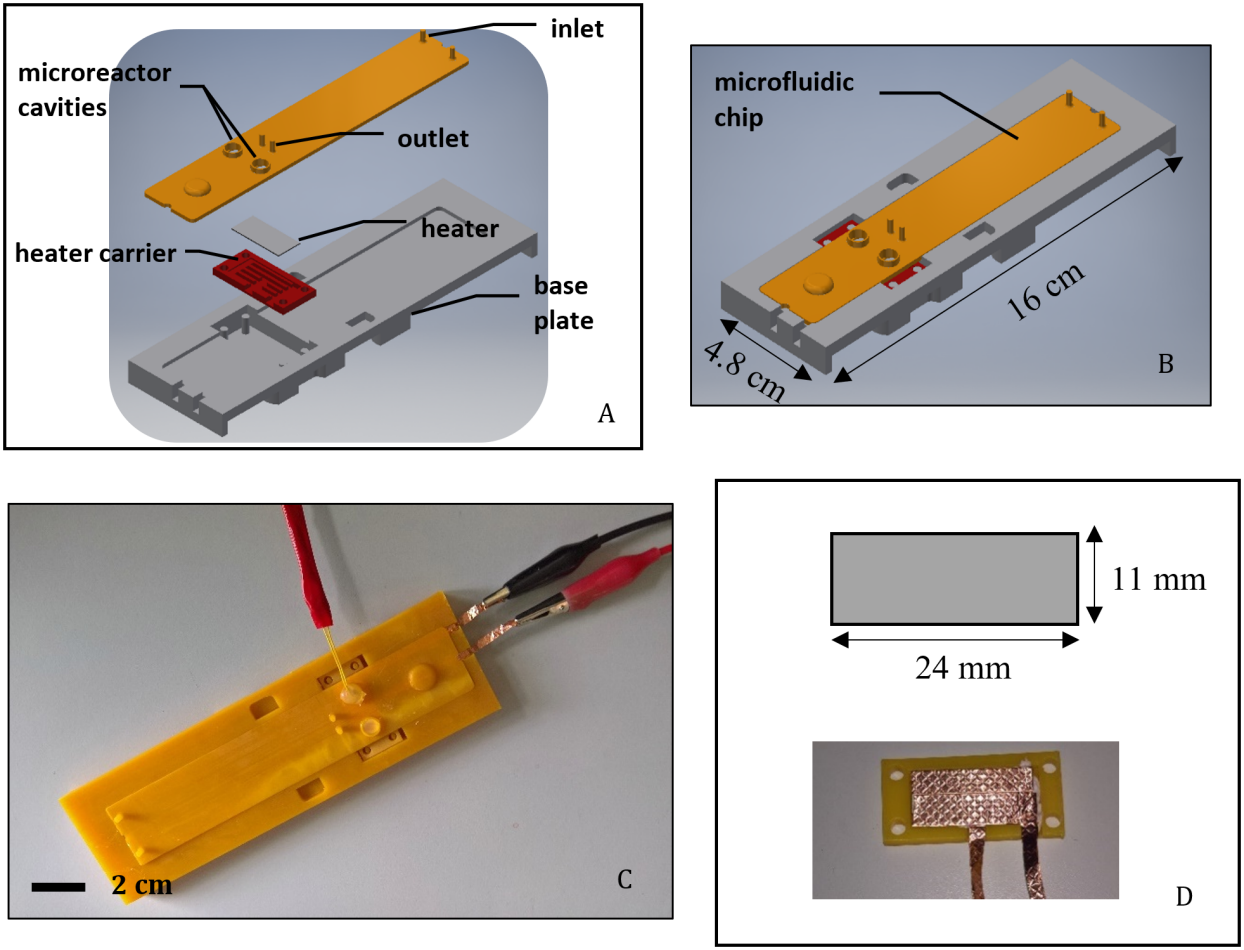
Experimental setup rendered in exploded and assembled view. The setup (A-B) contained a microfluidic chip with 2 microreactor cavities holding 0.1 ml liquid volume in total, and an integrated self-regulating microheating element (Heatron Inc. BM117-83) attached to a carrier (D). Fluidic inputs were connected to 3 ml syringes. The fully assembled setup for thermal characterization (C) was connected to 2xAAA batteries to power the heating element, and a temperature probe was fixed for temperature recording with thermoplastic adhesive in one of the reactor cavities.

BM117 batch self-regulating resistive heating elements (Fig 1D) provided by Heatron Inc. were PTC polymer heaters [24]. Two sets of heaters were provided from sample lots with differing electrical characteristics as shown in Table 1. The heater had a sandwich structure consisting of the resin sandwiched between 2 conductive films. The heater was contacted externally by adhesive copper tape and wires.

**Table 1:** Design parameters for self-regulating PTC polymer heaters from Heatron Inc.

Batch and lot nr.	Design input voltage [V]	Design base resistance [Ω] (+25% max. tolerance)	Design target temperature range [°C]
BM117-83-A	3	1.15	58–62
BM117-83-B	3	0.5	60–64

Different sample lots with overlapping target temperatures were chosen to find an ideal match for a better control precision than was possible by design.

### 2.2 Thermal modeling

Heat transfer analysis was based on finite element modelling. The proposed model was built to simulate temperature distribution in the steady state, given a known, experimentally characterized heating element and a 3D device geometry defined along with structural materials. The resistive heating element generates volumetric heat flux Q via Joule heating in the following form [25,26]:

$$\begin{array}{l} Q=J\cdot E\\ \nabla J=Q_{j}\\ J=\sigma E\\ E=-\nabla V. \end{array}$$ (1)

Where J, E, Q_j_, and V are the current density, electric field, current sources (sinks) and potential drop in the heating element, respectively. σ characterizes the temperature-dependent conductivity of the PTC polymer resin in the heating element. The generated heat is propagated in the device via the heat transfer equation assuming zero flow (during amplification the reaction volume is halted in the reaction chamber, no movement is permitted) in the following form [27]:

$$\rho C_{p}\nabla T-\nabla \cdot (k\nabla T)=Q$$ (2)

Where structural materials are characterized by their density ρ, specific heat capacity C_p_, absolute temperature T and thermal conductivity k. For solving the model, COMSOL^®^ Multiphysics (version 5.2) was used on a PC with a Core i5-4570 CPU and 16 GB RAM. The Heat Transfer and Electric Currents interfaces of COMSOL were used, coupled through the Joule Heating interface. Three-dimensional model geometry was imported from Autodesk Inventor. Contact films were not included in the model in order to save computational space and due to their small size (<0.1 mm thickness) in comparison with the rest of the model. Furthermore, the model was cut along its plane of symmetry (y-z) to save computational space. Boundary conditions and initial values were derived from the experimental setup (Table 2). The model was solved with the built-in Stationary solver of COMSOL. Material properties were derived from library values or manufacturer data sheets (Table 3). The temperature-dependent conductivity profile of the heating element was calculated from data provided by Heatron Inc. Convective, conductive and radiative heat losses were taken into account in the model. COMSOL generated a quadratic mesh with an average element quality (based on the well-known radius ratio method) of 0.68 and element size of 0.07 mm^3^.

**Table 2:** Boundary conditions and initial parameter values for the model.

Boundary condition	Boundary	Initial value (if applicable)
Ambient temperature	External boundaries	20°C
Ambient pressure (absolute)	External boundaries	1 atm
Electric potential	Heater (top)	3 V
Ground	Heater (bottom)	0 V
Convective heat loss	External boundaries	Not applicable
Electrical insulation	Heater boundaries except bottom and top	Not applicable
Symmetry	Symmetry plane	Not applicable
Joule heating (boundary)	Heater boundaries	Not applicable

**Table 3:** Material properties for the model.

Material	Density (ρ) [kg/m³]	Thermal conductivity (k) [W/(m·K)]	Specific heat capacity (C_p_) [J/kg·K]	Conductivity (σ) [S/m]
Polycarbonate plastic (frame and chip)	1200	0.14	1250	Not relevant
PTCR heater resin	1673	0.43	1000	0.8–0.05 (20–65°C range)
Air	1225	0.024	1000	Not relevant
Water	1000	0.6	4184	Not relevant

Please note that conductivity is only listed for materials that correspond to physical domains involved in the heating process.

### 2.3 Experimental setup for thermal characterization

The experimental setup was designed as a thermal mock-up for the isothermal nucleic acid amplification Lab-on-a-Chip system currently under development in our labs (Fig 1A–1C). The mock-up consisted of a microfluidic chip with an integrated microheating element on a carrier platform and a base plate. The chip’s body was 3D printed by an SLA 3D printing system (Envisiontec Perfactory XL), and the channels were sealed with a Greiner multiwell plate sealer film (P/N A5596 from Sigma-Aldrich). The fluidic chip contained 2 microreactor cavities with 0.1 ml total volume, as well as four fluidic connections (2 inlets, 2 outlets for degassing). The channel network contained a splitting element before the microreactor cavities. Liquid input (distilled water) was introduced through one of the input features using a 3 ml syringe, while the other input was sealed with an empty 3 ml syringe (0 ml air). The 50 μl microreactor cavities were sealed from above with thermoplastic adhesive, and a digital thermometer probe was inserted into one of them. The heating element was attached to a carrier plate. The heater was contacted on both sides by adhesive copper tape (3M-1245) and the tape was attached to wires extended beyond the baseplate. All components were clamped to a base plate. Above the heated area, an additional layer of polyurethane foam (ESD P/N 961.1000.10) was placed for insulation. Fluidic components were connected with plastic tubing.

### 2.4 Evaluation in isothermal NAAT

The experimental setup for testing the prototype as a NAAT system was based on the aforementioned Lab-on-a-Chip thermal mock-up without the temperature probe, sealed airtight. The reaction chambers of the chip were sealed by plastic caps and thermoplastic adhesive, and the inlet and outlet features were connected to 3 ml syringes by plastic tubing. Syringes connected to the outlet were filled with air to act as gas springs (to aid even splitting of the reaction volume), while one inlet syringe was left empty and the other contained the liquid master mix and sample, prepared outside the chip before testing. A LAMP (loop-mediated isothermal amplification) NAAT assay developed for chlamydia trachomatis (CT) was selected as the candidate for on-chip testing, and the assay was set up as demonstrated by Jevtuševskaja et al. [28] and Tulp et al. [29]. However, for our demonstration, template DNA (Serotype E; DSMZ; DSM 19131 at a concentration of 2000 copies per reaction) was used instead of DNA lysed from urine samples. The experiment was done in duplicate, in two identical Lab-on-a-Chip prototypes. Therefore, four equal volumes of liquid master mix and buffer were prepared in Eppendorf tubes, and mixed with the DNA samples, resulting in a total volume of 250 μl for each chip.

## 3 Results and discussion

### 3.1 Experimental evaluation of heating element samples

Before thermal characterization, each heating element was characterized electrically using an Agilent 34410A digital multimeter. Table 4 summarizes base resistance values recorded at room temperature, as well as calculated steady-state resistances. For steady-state resistance calculations, heaters were powered up from an Agilent E3631A DC Power Supply and the input current as well as voltage values were recorded. The steady-state resistance value was calculated from the minimum input current and the constant voltage input.

**Table 4:** A summary of samples of BM117-83 batch self-regulating resistive heating elements.

Heater (BM117-83-)	Resistance [Ω] at RT (22–24 degC)	Steady-state resistance [Ω] (heated up)
A20	1.15	12.57
A24	1.12	12.39
B1	0.5	15.08
B12	0.52	14.46

2 samples were picked from both sample lots marked by A and B, with lower and higher targeted set temperatures. Samples were characterized with respect to their base resistance. Steady-state resistance was calculated based on input current and voltage after allowing the heating element to reach steady-state when powered.

For the thermal characterization, the experimental setup was assembled as mentioned in section 3.3. Each experiment was conducted with a fresh set of 2pcs. alkaline AAA batteries. The microreactor cavities were filled with liquid and contacts were connected. Recording was performed for 30 minutes with each measurement. Between experiments, the setup was allowed to cool down to room temperature. Room temperature for the experiments was defined as 20–25°C and monitored with a digital thermometer (TENMA 72–7715 with K-type thermocouples). For temperature recording, 10K NTC Thermistors were used (Vishay BC NTCLE300E3103SB), recording was done by a digital multimeter (Agilent 34410A) using a scripted computer interface through MATLAB.

**Fig 2:**
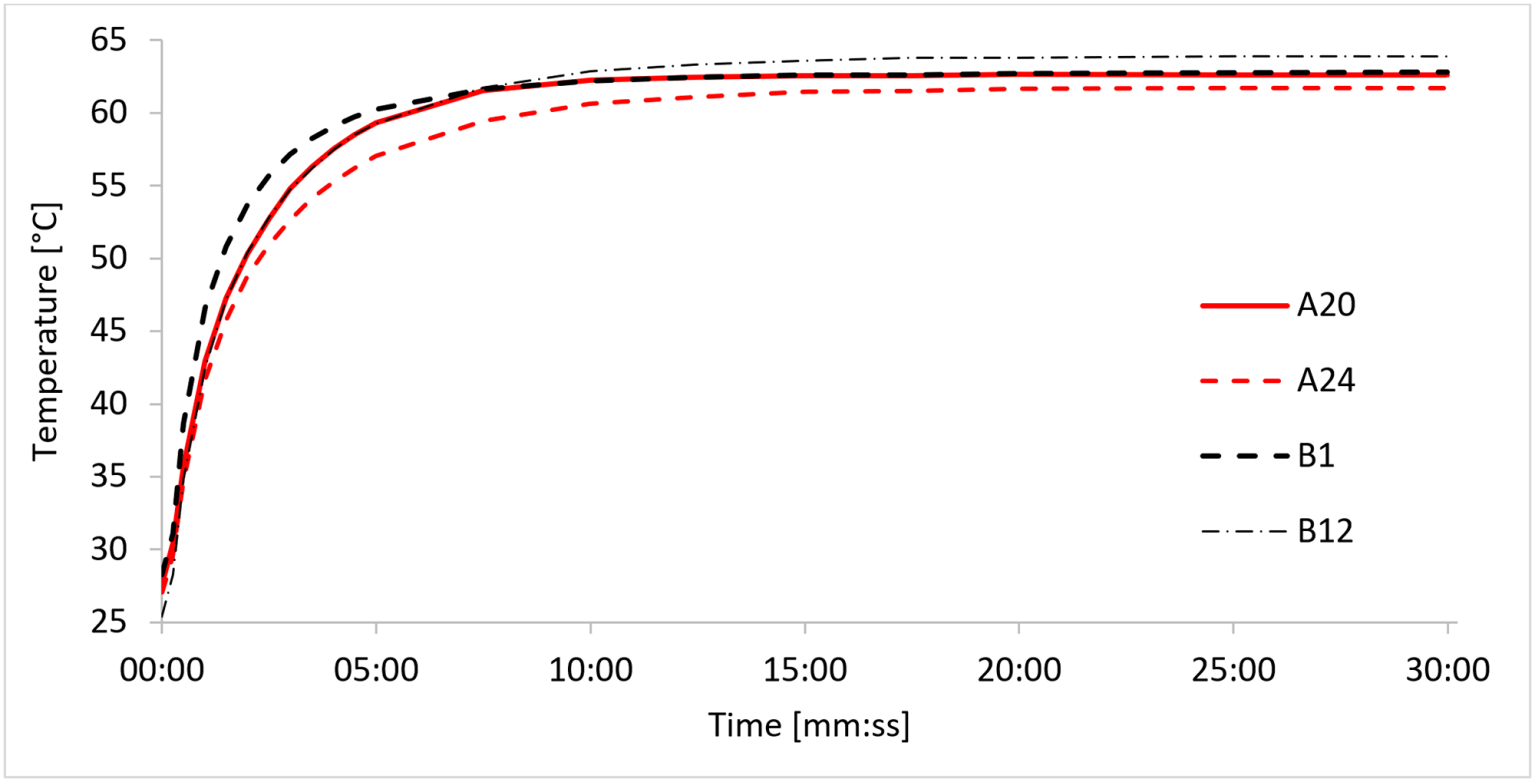
Heating element sample performance in the tested Lab-on-a-Chip experimental setup. Although differences were minor, heating element BM117-83-B1 was the most suitable candidate from the tested samples, both with respect to steady-state temperature and rise times achieved during the test.

Recorded temperatures (S1 Dataset) are shown in Fig 2 for each tested heating element sample. The tested samples were able to elevate temperatures in the test setup to 61.5–64°C and hold for 25 minutes. Samples A20, A24 and B1 conformed to the temperature criterion. However, only sample B1 conformed to the time criterion of 5 minutes to reach target range. Thus, sample B1 was demonstrated to be the optimal candidate for isothermal nucleic acid amplification tests.

### 3.2 Evaluation by thermal modelling

**Fig 3:**
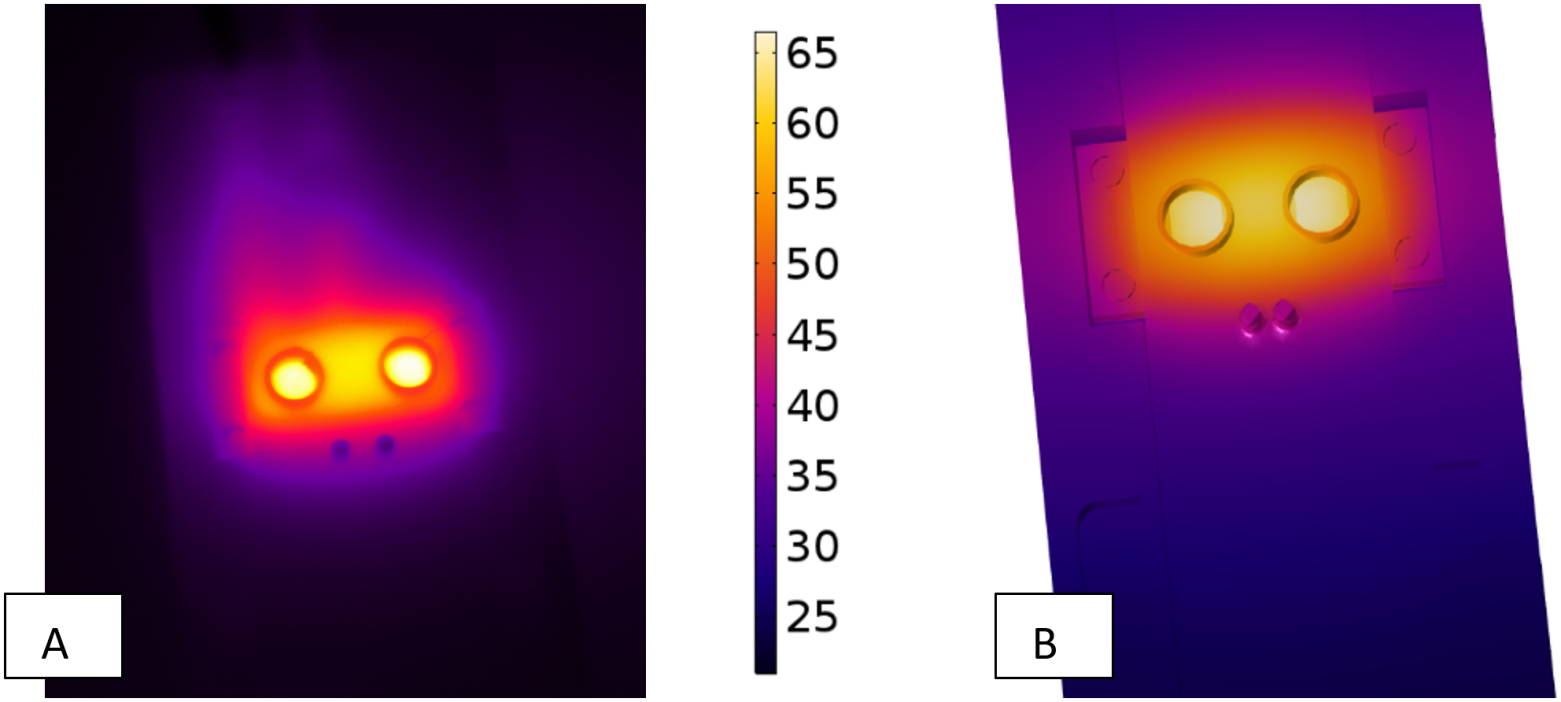
Comparison of the simulated temperature distribution to the thermal image of the physical prototype. Both simulated (A) and experimentally recorded (C) images are shown in steady state. Heating element BM117-83-B1 was selected as the candidate for comparison due to its favorable performance. Its temperature-dependent resistivity profile was fed into the model. In both the physical and simulated prototype, temperature probes were placed in the same spot (see Fig 1C) and recorded steady-state temperatures compared. The model estimated recorded temperatures with less than 0.2°C absolute error.

The model was set up for heater sample BM117-83-B1 for the evaluation. Material properties were defined by library values from COMSOL^®^ Multiphysics or manually based on data from Heatron Inc. Average solution time was 40 s (±3.5). For a side-by-side comparison of simulated and recorded temperature maps (Fig 3), infrared images of the experimental setup were captured by a Jenoptik VarioCAM 384 HiRes IR camera. A temperature probe (domain point probe to extract values) was defined in COMSOL at the location of the physical temperature probe in the experimental setup. Comparing the two probes indicated that the model estimated recorded steady-state temperatures with an absolute error of 0.16°C.

**Fig 4:**
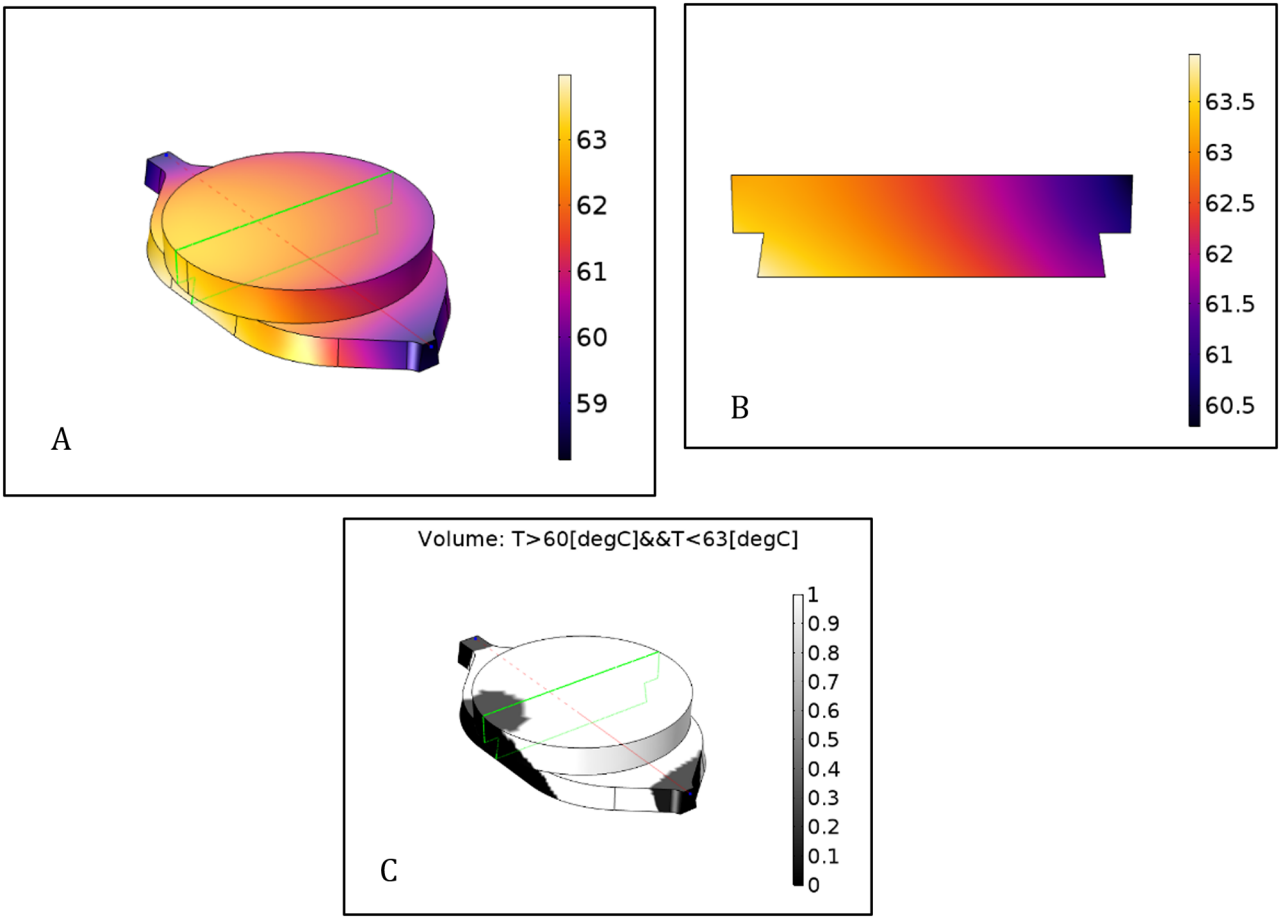
Color maps showing the spatial distribution of temperatures in the microreactor cavity. The temperature distribution map (A) indicated a gradient from the inner towards the outer reactor wall, however, the cross-section (cut plane marked in green) indicated that the microreactor temperature was within the specified target range of 60–63°C (B). Calculations based on model outputs indicated that ≈85% of the reaction volume was within the target range in steady state (C).

A volumetric temperature condition was defined for the reaction chamber (*T* ∈ [60;63°*C*]) in the model and the reaction volume in the desired target range was calculated. In the simulated setup, results indicated that in steady-state, an estimated 84.6% of the whole reaction volume (≈42 μl per chamber) was within the desired target temperature range (Fig 4C), which was adequate to run an isothermal amplification reaction that produced enough amplicons for a positive result, as demonstrated in Section 4.3. Finite element modelling allows the extrapolation of temperature distribution in areas not measureable by physical means and helps gain insight into thermal phenomena beyond the level achievable by experimentation alone. Visualizing temperature distributions over the whole microreactor volume (Fig 4A) and its cross-section (Fig 4B) indicated a temperature gradient from the inner to the outer (closer to the edge of the chip) reactor wall. This will be taken into account in future device geometry designs by adding additional insulation to the outer reactor wall.

### 3.3 Evaluation as NAAT

**Fig 5:**
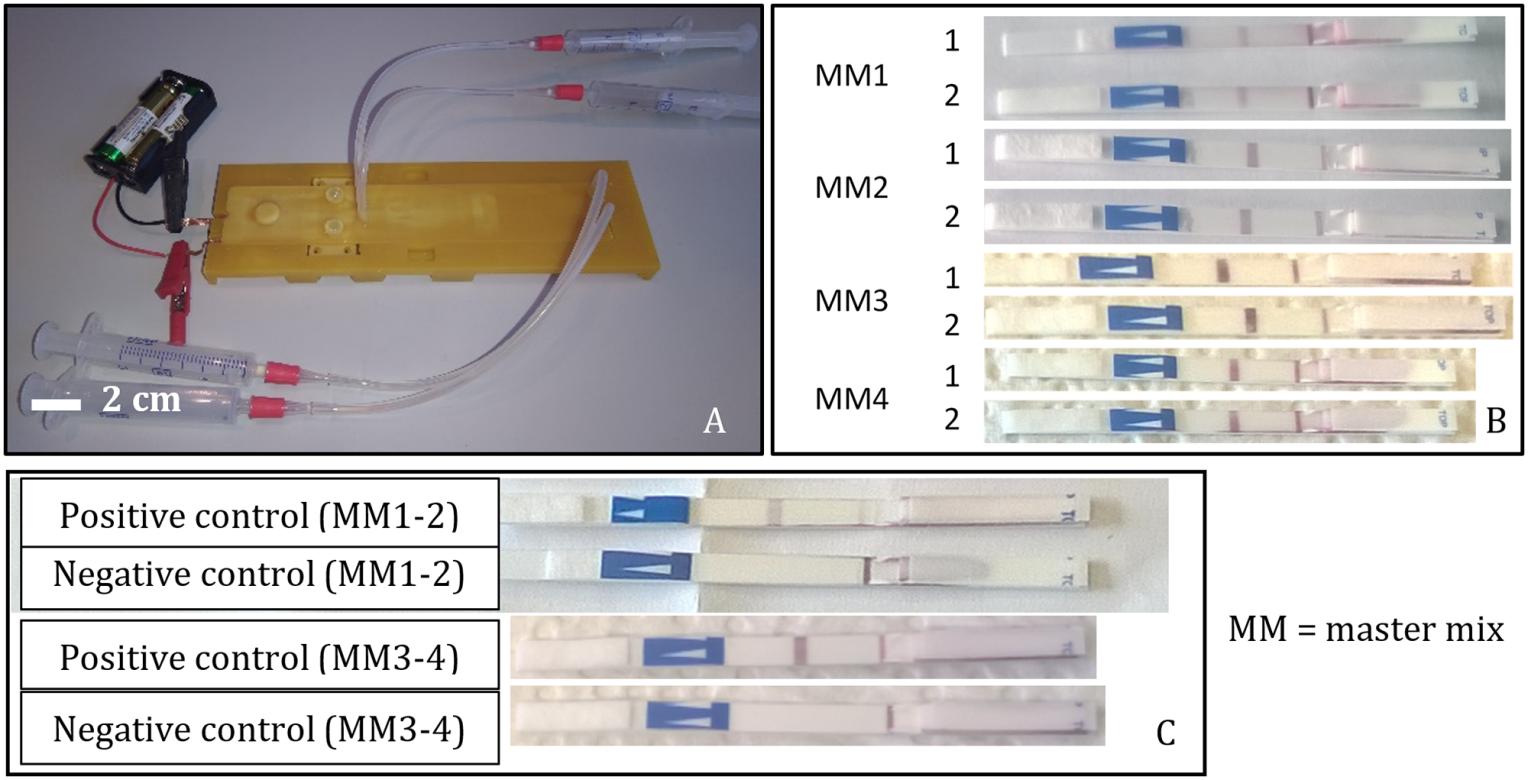
Evaluation of the self-regulating resistive heating element with an on-chip isothermal nucleic acid amplification protocol. Two Lab-on-a-Chip prototypes with two reaction chambers were prepared along with reaction volumes including samples and master mix separately (A). After 30 minutes of incubation with the heating element, reaction volumes were extracted into Eppendorf tubes and lateral flow strips were added to detect amplicons. The experiment was repeated twice with two chips. Results indicated a successful amplification for all 8 reaction volumes (B). The result was confirmed by separately performing LAMP for a positive and negative control in Eppendorf tubes (C).

After sealing the chip and filling in the reaction volume, batteries were connected to the heating element (Fig 5A). To prevent potential cross-contamination, the experimental setup was sealed in a plastic bag and incubation was performed under a lateral flow hood. Incubation time was 30 minutes in total, including the 5 minutes allowed for the heating element to reach steady state. The test protocol was repeated four times in total, twice for both Lab-on-a-Chip prototypes, for both reaction chambers. After incubation, reaction volumes were extracted and pipetted into Eppendorf tubes, into which lateral flow strips were placed to detect amplicons. Results indicated a successful amplification in all 8 reaction chambers tested (Fig 5B). Parallel to the experiments performed on chip, separate positive and negative controls (containing the master mix only) were prepared in Eppendorf tubes and the amplification was successfully performed in a heating block, confirming on-chip test results (Fig 5C).

## 4 Conclusions

We demonstrated a self-regulating resistive heating solution for disposable LoC NAAT devices. 4 heating element samples (Fig 1D) were provided by Heatron Inc. with different temperature-dependent resistivity profiles (Table 4) that matched the lower and upper end of the targeted 60–63°C temperature range. A thermal mock-up was made for a LoC NAAT device with 2 microreactor chambers, 50μl volume each (Fig 1A–1C). Each heating element sample was characterized by transient thermal analyses: a temperature recording of over 30 minutes of operation. Sample BM117-83-B1 was demonstrated capable of heating the liquid in the reaction chamber to the range 60–63°C in 5 minutes and holding for 25 minutes (Fig 2). Further thermal analysis was performed using finite element analysis of heat transfer. Steady state simulation results indicated that 85% of the reaction volume was kept in the specified target temperature range (Fig 4). Finally, the proposed integrated heating solution was demonstrated in a LoC NAAT prototype to be capable of supporting a LAMP assay for the detection of Chlamydia Trachomatis template DNA (Fig 5).

## Supporting information

S1 Dataset Temperature data for BM117-83-A and -B lot PTCR polymer resin heater samples. (PDF)

## References

[1] Craw P, Balachandran W. Isothermal nucleic acid amplification technologies for point-of-care diagnostics: a critical review. Lab Chip. 2012;12: 2469 doi: 10.1039/c2lc40100b 22592150 10.1039/c2lc40100b

[2] Notomi T. Loop-mediated isothermal amplification of DNA. Nucleic Acids Res. 2000;28: 63e–63. doi: 10.1093/nar/28.12.e63 10.1093/nar/28.12.e63 PMC102748 10871386

[3] Mori Y, Notomi T. Loop-mediated isothermal amplification (LAMP): a rapid, accurate, and cost-effective diagnostic method for infectious diseases. J Infect Chemother. 2009;15: 62–69. doi: 10.1007/s10156-009-0669-9 19396514 10.1007/s10156-009-0669-9 PMC7087713

[4] Asiello PJ, Baeumner AJ. Miniaturized isothermal nucleic acid amplification, a review. Lab Chip. 2011;11: 1420 doi: 10.1039/c0lc00666a 21387067 10.1039/c0lc00666a

[5] Mark D, Haeberle S, Roth G, Von Stetten F, Zengerle R. Microfluidic Lab-on-a-Chip Platforms: Requirements, Characteristics and Applications In: Kakaç S, Kosoy B, Li D, Pramuanjaroenkij A, editors. Microfluidics Based Microsystems. Dordrecht: Springer Netherlands; 2010 pp. 305–376. http://link.springer.com/10.1007/978-90-481-9029-4_17

[6] Velve Casquillas G, Fu C, Le Berre M, Cramer J, Meance S, Plecis A, et al Fast microfluidic temperature control for high resolution live cell imaging. Lab Chip. 2011;11: 484–489. doi: 10.1039/c0lc00222d 21103458 10.1039/c0lc00222d

[7] Velve-Casquillas G, Costa J, Carlier-Grynkorn F, Mayeux A, Tran PT. A Fast Microfluidic Temperature Control Device for Studying Microtubule Dynamics in Fission Yeast. Methods in Cell Biology. Elsevier; 2010 pp. 185–201. doi: 10.1016/S0091-679X(10)97011-8 20719272 10.1016/S0091-679X(10)97011-8 PMC2997723

[8] Maltezos G, Johnston M, Taganov K, Srichantaratsamee C, Gorman J, Baltimore D, et al Exploring the limits of ultrafast polymerase chain reaction using liquid for thermal heat exchange: A proof of principle. Appl Phys Lett. 2010;97: 264101 doi: 10.1063/1.3530452 21267083 10.1063/1.3530452 PMC3026011

[9] Mao H, Yang T, Cremer PS. A Microfluidic Device with a Linear Temperature Gradient for Parallel and Combinatorial Measurements. J Am Chem Soc. 2002;124: 4432–4435. doi: 10.1021/ja017625x 11960472 10.1021/ja017625x

[10] Scorzoni A, Tavernelli M, Placidi P, Zampolli S. Thermal Modeling and Characterization of a Thin-Film Heater on Glass Substrate for Lab-on-Chip Applications. IEEE Trans Instrum Meas. 2015;64: 1098–1098. doi: 10.1109/TIM.2014.2364697

[11] Moschou D, Vourdas N, Kokkoris G, Papadakis G, Parthenios J, Chatzandroulis S, et al All-plastic, low-power, disposable, continuous-flow PCR chip with integrated microheaters for rapid DNA amplification. Sens Actuators B Chem. 2014;199: 470–478. doi: 10.1016/j.snb.2014.04.007

[12] Jiao Z, Huang X, Nguyen N-T, Abgrall P. Thermocapillary actuation of droplet in a planar microchannel. Microfluid Nanofluidics. 2008;5: 205–214. doi: 10.1007/s10404-007-0235-7

[13] Darhuber AA, Valentino JP, Troian SM, Wagner S. Thermocapillary actuation of droplets on chemically patterned surfaces by programmable microheater arrays. J Microelectromechanical Syst. 2003;12: 873–879. doi: 10.1109/JMEMS.2003.820267

[14] Selva B, Miralles V, Cantat I, Jullien M-C. Thermocapillary actuation by optimized resistor pattern: bubbles and droplets displacing, switching and trapping. Lab Chip. 2010;10: 1835 doi: 10.1039/c001900c 20445893 10.1039/c001900c

[15] Wyzkiewicz I, Grabowska I, Chudy M, Brzozka Z, Jakubowska M, Wisniewski T, et al Self-regulating heater for microfluidic reactors. Sens Actuators B Chem. 2006;114: 893–896. doi: 10.1016/j.snb.2005.08.028

[16] Pardy T, Rang T, Tulp I. Development of Temperature Control Solutions for Non-Instrumented Nucleic Acid Amplification Tests (NINAAT). Micromachines. 2017;8: 180 doi: 10.3390/mi8060180

[17] Pardy T, Rang T, Tulp I. Modelling and experimental characterisation of self-regulating resistive heating elements for disposable medical diagnostics devices WIT Transactions on Engineering Sciences. Valencia, Spain: WIT Press; 2015 pp. 263–271. doi: 10.2495/MC150241

[18] Grabowska I, Stadnik D, Chudy M, Dybko A, Brzozka Z. Architecture and method of fabrication PDMS system for uric acid determination. Sens Actuators B Chem. 2007;121: 445–451. doi: 10.1016/j.snb.2006.04.101

[19] Streit P, Nestler J, Schulze R, Shaporin A, Otto T. Investigation on the temperature distribution of integrated heater configurations in a Lab-on-a-Chip system. IEEE; 2017 pp. 1–8. doi: 10.1109/EuroSimE.2017.7926229

[20] Buser JR, Diesburg S, Singleton J, Guelig D, Bishop JD, Zentner C, et al Precision chemical heating for diagnostic devices. Lab Chip. 2015;15: 4423–4432. doi: 10.1039/c5lc01053e 26503640 10.1039/c5lc01053e PMC10249953

[21] Weigl B, Domingo G, LaBarre P, Gerlach J. Towards non- and minimally instrumented, microfluidics-based diagnostic devices. Lab Chip. 2008;8: 1999 doi: 10.1039/b811314a 19023463 10.1039/b811314a PMC2776042

[22] Singleton J, Zentner C, Buser J, Yager P, LaBarre P, Weigl BH. Instrument-free exothermic heating with phase change temperature control for paper microfluidic devices. In: Becker H, Gray BL, editors. 2013 p. 86150R doi: 10.1117/12.2005928 25426269 10.1117/12.2005928 PMC4241343

[23] Craw P, Balachandran W. Isothermal nucleic acid amplification technologies for point-of-care diagnostics: a critical review. Lab Chip. 2012;12: 2469 doi: 10.1039/c2lc40100b 22592150 10.1039/c2lc40100b

[24] Carmona F, Maire J, Septier H, Canet R, Delhaes P. Material having a resistivity with a positive temperature coefficient. US4966729 A, 1990.

[25] von Meier A. Electric power systems: a conceptual introduction. Hoboken, NJ: Wiley-Interscience [u.a.]; 2006.

[26] Griffiths DJ. Introduction to electrodynamics. Prentice Hall; 1999.

[27] Kandlikar SG, editor. Heat transfer and fluid flow in minichannels and microchannels. 1. ed. Amsterdam: Elsevier; 2006.

[28] Jevtuševskaja J, Uusna J, Andresen L, Krõlov K, Laanpere M, Grellier T, et al Combination with antimicrobial peptide lyses improves loop-mediated isothermal amplification based method for Chlamydia trachomatis detection directly in urine sample. BMC Infect Dis. 2016;16 doi: 10.1186/s12879-016-1674-0 27412444 10.1186/s12879-016-1674-0 PMC4944247

[29] Indrek Tulp, Katrin Krolov, Marko Lehes, Ulo Langel. Method and its compositions for detection of nucleic acid target from biological samples and body fluids. US20150322493A1.

