# Supplementary material for: Integrated self-regulating resistive heating for isothermal nucleic acid amplification tests (NAAT) in Lab-on-a-Chip (LoC) devices
Source: PLoS One. 2017 Dec 21;12(12):e0189968. doi: 10.1371/journal.pone.0189968 (PMC5739446; doi:10.1371/journal.pone.0189968)
Supplement: S1 Dataset — (PDF) [file pone.0189968.s001.pdf]

	A20				A24		
Time	mean	stdev		Time	mean	stdev	
00:00	27.54534	0.054658		00:00	27.03754	0.062463	
00:15	30.4156	1.315601		00:15	29.47947	1.179471	
00:30	35.67486	1.074862		00:30	34.57639	1.576386	
01:00	42.96424	0.664241		01:00	41.59894	1.19894	
01:30	47.24622	0.846223		01:30	45.81703	1.417028	
02:00	50.35566	1.155659		02:00	48.75002	1.650016	
02:30	52.74558	1.445584		02:30	50.92509	1.925086	
03:00	54.77851	1.578507		03:00	52.69164	2.091643	
03:30	56.28063	1.680628		03:30	54.08713	2.187128	
04:00	57.51339	1.71339		04:00	55.23017	2.230168	
04:30	58.51951	1.719514		04:30	56.20554	2.205538	
05:00	59.29608	1.69608		05:00	57.04954	2.14954	
07:30	61.50092	1.400923		07:30	59.46109	1.961085	
10:00	62.24709	1.247093		10:00	60.64133	1.74133	
12:30	62.44808	1.248081		12:30	61.10563	1.705626	
15:00	62.53844	1.238441		15:00	61.43773	1.637733	
17:30	62.55819	1.258187		17:30	61.51062	1.710625	
20:00	62.62534	1.225337		20:00	61.65082	1.650824	
25:00	62.5926	1.292603		25:00	61.68015	1.680151	
30:00	62.59393	1.293929		30:00	61.68651	1.686512	
steady-state	62.60396			steady-state	61.6725		
stdev(steady-state)	0.015128			stdev(steady-state)	0.015543		

	B1				B12	
Time	mean	stdev		Time	mean	stdev
00:00	28.26482	0.164819		00:00	25.37058	0.070578
00:15	31.09484	1.994841		00:15	28.26799	1.867995
00:30	38.6409	2.540904		00:30	34.79155	0.891549
01:00	46.57157	3.271569		01:00	42.4805	0.480499
01:30	50.81104	3.41104		01:30	47.157	0.556996
02:00	53.70709	3.507093		02:00	50.41966	0.71966
02:30	55.70914	3.609136		02:30	52.87744	0.97744
03:00	57.15757	3.657571		03:00	54.74484	1.144838
03:30	58.25733	3.65733		03:30	56.19487	1.294871
04:00	59.06617	3.666167		04:00	57.47642	1.276416
04:30	59.71905	3.619051		04:30	58.49096	1.290962
05:00	60.2534	3.553405		05:00	59.28735	1.287346
07:30	61.62897	3.42897		07:30	61.75129	1.051288
10:00	62.17726	3.377264		10:00	62.87768	0.877683
12:30	62.44027	3.340272		12:30	63.34414	0.844145
15:00	62.58396	3.283964		15:00	63.59991	0.799905
17:30	62.61792	3.31792		17:30	63.75541	0.755411
20:00	62.69634	3.296336		20:00	63.78857	0.788566
25:00	62.77192	3.271924		25:00	63.88105	0.781047
30:00	62.80145	3.301451		30:00	63.89503	0.795033
steady-state	62.75657			steady-state	63.85488	
stdev(steady-state)	0.044265			stdev(steady-state)	0.047239	
Simulated steady-state	62.599					
Absolute error:	0.15757					
